# Lifestyle, nutrition and breast cancer: facts and presumptions for consideration

**DOI:** 10.3332/ecancer.2015.557

**Published:** 2015-07-23

**Authors:** Krizia Ferrini, Francesca Ghelfi, Roberta Mannucci, Lucilla Titta

**Affiliations:** 1 SmartFood Program, European Institute of Oncology, Milan 20141, Italy; 2 Università degli Studi di Pavia, 27100, Italy; 3 Università degli Studi di Parma, 43121, Italy

**Keywords:** breast cancer, lifestyle, nutrition, cancer prevention, body weight

## Abstract

Breast cancer is the most common cancer in women worldwide, and the high incidence of this cancer coupled with improvements in initial treatments has led to an ever-increasing number of breast cancer survivors. Among the prospective epidemiological studies on diet and breast cancer incidence and recurrence, to date, there is no association that is strong, reproducible and statistically significant, with the exception of alcohol intake, overweight, and weight gain. Nevertheless, many beliefs about food and breast cancer persist in the absence of supporting scientific evidence.

After a comprehensive review regarding the role of lifestyle on breast cancer outcomes and a thorough study of the dissemination field including mass media, clinical institutions, and academic figures, we briefly reported the most common presumptions and also facts from the literature regarding lifestyle, nutrition, and breast cancer.

The randomised controlled trial is the best study-design that could provide direct evidence of a causal relationship; however, there are methodological difficulties in applying and maintaining a lifestyle intervention for a sufficient period; consequently, there is a lack of this type of study in the literature. Instead, it is possible to obtain indirect evidence from observational prospective studies. In this article, it becomes clear that for now the best advice for women’s health is to follow the World Cancer Research Fund/American Institute of Cancer Research (WCRF/AICR) recommendations on diet, nutrition, physical activity, and weight management for cancer prevention, because they are associated with a lower risk of developing most types of cancer, including breast cancer. Despite current awareness of the role of nutrition in cancer outcomes, there is inadequate translation from research findings into clinical practice. We suggest the establishment of a multidisciplinary research consortium to demonstrate the real power of lifestyle interventions.

## Introduction

Breast cancer is the most common cancer in women, and its frequency is rising in countries with low and middle incomes. Approximately, one in eight women worldwide will develop breast cancer during her life and breast cancer is the first-leading cause of cancer deaths among women [1].

A number of risk factors have been identified in the pathogenesis of breast tumours; among these, a great number are linked to nutrition and life-style (e.g., alcohol consumption, obesity, and eating patterns) [2].

The influence of diet on mammary carcinogenesis has been clearly demonstrated in animal models. Obesity induced by high-fat diets increases the risk of several cancers, including breast cancer in several animal species [3]. On the contrary, soybean products act as cancer preventive agents in rodents and other animals [4].

Epidemiological studies provide clear evidence of association between breast cancer incidence, mortality, and dietary patterns or dietary constituents in humans, but frequently there is a discrepancy between the studies on human populations and investigations performed in more controlled conditions, such as cellular or animal studies. Many other variables, such as race, age, menopausal status, onset of puberty and the number of pregnancies, are in fact associated with diet and lifestyle in humans, acting as confounding factors [5].

Among the prospective epidemiological studies on diet and breast cancer incidence, to date, there is no association, that is strong, reproducible, and statistically significant, with the exception of alcohol intake, overweight, and weight gain [6]. Over the past decade, there have been fundamental advances in breast cancer genomics.

As nutrition deeply impacts on genome, it is critical to investigate this interaction. Nutrigenomics can clarify how genetic and epigenetic variations, which control gene expression, are influenced by nutrients and how development, progression, and recurrence of cancer are modified by foods [7].

In this review, we will summarise the current scientific evidence regarding the association between food, lifestyle, and breast cancer risk in women.

## Evidence of body weight and breast cancer

Increased body mass index (BMI) is associated with a significant increase in the risk of breast cancer, although with some differences in age and menopausal status. The association between being overweight (defined as a BMI of 25 to 29.9 kg/m^2^) or obese (BMI of 30 kg/m^2^ or greater) and breast cancer incidence has been found in many studies. Most studies and meta-analysis showed an increased risk of breast cancer for postmenopausal women and an inverse association between obesity and survival after breast cancer [8]. Controversial data exist in premenopausal subjects. Several studies, including the Million Women Study, report that the risk of breast cancer decreases with increasing BMI with a linear relationship along the BMI scale starting from 20 kg/m^2^ [9]. On the contrary, obesity increases breast cancer incidence in premenopausal women in the Asian population [10]. Furthermore, abdominal fatness is closely associated with premenopausal cancer risk [11]. A meta-analysis showed that the premenopausal breast cancer risk is 79% higher in women with the highest waist height ratio (WHR) compared with those with the lowest WHR [10].

Obesity, abdominal obesity in particular, has been associated with the production of adipokines in fat tissues [12], which, in turn, causes insulin insensitivity in peripheral tissues [13]. Insulin resistance leads to elevated blood glucose levels, which stimulate the production of insulin and insulin-like growth factors (IGFs) from pancreatic β-cells [3]. Insulin-like growth factor 1 (IGF-1) induces the activation of oestrogen receptor-α (ERα) in a ligand-independent manner through the involvement of mitogen-activated protein kinase (MAPK) signalling. ERα is responsible for many of the effects of oestrogen on normal and cancerous breast tissue, through ligand-activated transcriptional regulation (genomic actions) and by acting as a component of membrane and cytoplasmic signalling cascades (non-genomic actions) [14].

Body fat increases oestrogen production through the aromatisation of steroidal precursors of both ovarian and adrenal origin. Tumorigenic properties of oestrogen are regulated through the oestrogen receptor (ER), making the understanding of the mechanisms that activate this receptor highly relevant. In addition to oestrogen activating the ER, other growth factor pathways, such as IGFs, can activate the ER.

Recently, a possible interaction between leptin and insulin, and obesity-related markers of inflammation has been linked to breast cancer outcomes. Numerous studies have examined the relationship between obesity and prognosis in breast cancer: the majority of them demonstrate an inverse association between obesity and survival after breast cancer [15, 16]. However, it is still unknown whether intentional weight loss improves outcomes for women with breast cancer [17] and the number of trials of weight loss interventions in these patients, while increasing, is still small [18].

Recently, the International Agency for Research on Cancer (IARC) and the World Cancer Research Fund (WCRF) have remarked that diet strongly influences cancer prevention, disease development, treatment tolerance, and cancer recurrence and also provide advice and recommendations for body weight management in breast cancer primary and secondary prevention [19, 20].

## Evidence of alcohol consumption and breast cancer

A number of epidemiological studies have provided convincing evidence that alcohol consumption is an important risk factor for the incidence and mortality of breast cancer [21, 22].

A pooled analysis of six prospective cohort studies showed that alcohol consumption is associated with a linear increase in breast cancer incidence. The adjusted relative risk for total alcohol intakes of 30–60 g/day (about 2–5 drinks) versus non-drinkers was 1.41 (95% CI: 1.18–1.69) [23]. Furthermore, the Million Women Study showed that the relative risk of developing breast cancer increases by 7.1% for each 10 g/day of alcohol [24]. The relationship between breast cancer and alcohol was confirmed by the reanalysis of 53 epidemiological studies performed by Hamajima *et al* [22].

Ferrari *et al* collected data on 380 of 395 men and women (among whom 20,453 fatal events occurred) for which information on lifetime alcohol use was available, allowing separate consideration of former drinkers from lifetime abstainers. Lifetime average alcohol use was strongly associated with total mortality, in that heavy drinkers (≥30 g/day) had notably higher mortality rates than did light-to-moderate drinkers (0.1–4.9 g/day), a pattern that was consistently apparent among female and male study participants [21].

The mechanisms of carcinogenesis induced by alcohol on breast cancer are still unknown, but there is accumulating evidence that regular intakes of moderate amounts of alcohol affect sex hormone levels in premenopausal and postmenopausal women [25]. Several lines of evidence indicate that acetaldehyde, a product of alcohol metabolism, might play a role in alcohol-related carcinogenic effects in different target tissues [7, 22].

As alcohol is a lipid solvent, the substance could modify cell membranes enhancing cellular permeability to carcinogens. Furthermore, alcohol can modulate their metabolism, enhance the production of reactive oxygen species (ROS), and inhibit deoxy ribonucleic acid (DNA) repair [26].

The role of alcohol intake on breast cancer survival was investigated in The Life After Cancer Epidemiology (LACE) Study, a cohort of 2,321 early-stage breast cancer survivors designed to examine modifiable lifestyle predictors of recurrence, survival, and quality of life. Results suggested that alcohol consumption equivalent to 3–4 standard drinks or more per week was associated with the risk of breast cancer recurrence, particularly among postmenopausal and overweight/obese women [27].

Alcohol consumption also impairs folate metabolism and may alter gene methylation since folate plays a pivotal role in DNA methylation [7].

The analysis of methylation of a cluster of cancer-related genes has revealed that alcohol consumption is associated with a specific pattern of DNA methylation, thus suggesting that alcohol produces specific epigenetic changes.

## Evidence of lifestyle and breast cancer

Many behavioural choices during life are likely to enhance the risk of developing breast cancer. There is strong scientific evidence that not having children or having them after 30 years, avoiding breast feeding, prolonged use of the contraceptive pill, and having hormone replacement therapy after menopause all increase the incidence of mammary tumours. Other aspects of lifestyle have also been investigated. Among these, physical activity is associated with lower risk of invasive breast cancer [28]. However, breast cancers are heterogeneous for histopathology, hormone receptor status, and gene expression profile, and whether increased physical activity offers a true protection in all cases, is controversial. A considerable number of studies have been conducted worldwide on physical activity and breast cancer risk. On average, a 20–25% reduction in breast cancer risk has been observed among physically active women in comparison to the least active ones [29].

These results were confirmed in the recent European Prospective Investigation into Cancer and Nutrition (EPIC) study which demonstrated that moderate and high physical activity are associated with modest reduction in breast cancer risk [30]. According to these results, the Breast Cancer Report of WCRF and American Institute for Cancer Research (AICR) states that the evidence for reduction in cancer incidence in physically active women is probable [31]. A number of studies deal with the relationships between dietary habits and breast cancer incidence and mortality.

In evaluating the impact of diet and physical activity on cancer progression and survival, evidence from prospective observational studies suggests that physical activity and diet may be associated with improved cancer progression and recurrence [32].

In particular, a review by Davies *et al* suggests that a low-fat, high-fibre diet might be protective against breast cancer recurrence and progression; in this review, a large prospective study (*n* = 90,655) found that dietary fat increases the risk of recurrence or death in premenopausal women with breast cancer [33]. Any findings from observational studies need to be interpreted with caution and findings from observational studies need to be evaluated against findings from the more rigorous randomised controlled trials (RCTs), like the women’s intervention nutrition study (WINS) *n* = 2,437, a large-scale multicentre RCT which found a protective effect of a reduced fat diet. This protective effect was more evident in women with hormone-receptor-negative breast cancer [2].

Pierce *et al* have also shown that patients that consume a healthy diet and are physically active may increase their years of survival after diagnosis of breast cancer and those who reported eating a minimum of 5 vegetable and fruit servings daily and performing weekly physical activity equivalent to 30 minutes of walking at a moderate pace for 6 days a week had a higher 10-year survival rate than those who did not adhere to these lifestyle practices [34].

Spark *et al* evaluated postintervention maintenance of physical activity and dietary behaviour change outcomes; their findings suggest successful maintenance was more common in trials using a theoretical model for intervention development that included behavioural change strategies [35].

Due to these positive results, many public health agencies tend to promote a healthy lifestyle focused on reducing adiposity through a low-fat, high-fibre diet and regular physical activity. Among these are the WCRF/AICR (2007), the British Association of Sport and Exercise Sciences (2011), the Department of Health, Physical Activity Health Improvement, and Protection (UK) (2011), and the American College of Sports Medicine Roundtable on Exercise Guidelines for Cancer Survivors (2010) [32].

## Presumptions of a plant-based diet and the risk of breast cancer

Substances contained in vegetable foods are studied as possible ways to prevent cancer, since a number of studies have suggested that people who eat more fruit and vegetables are less likely develop cancer. Although there is evidence to show that a diet rich in plant foods may be protective against cancer risk and provide many health benefits, published data are not univocal [36]; in particular, the analysis of the correlation between diet and breast cancer is still a controversial issue.

By the 1980s, most reports dealing with the prevention of chronic diseases recommended relatively high intakes of vegetables and fruit, either because these foods were seen as nourishing substitutes for energy-dense fatty or sugary foods, or else because they were identified as positively protective agents against cardiovascular diseases. Evidence that vegetables and fruit might be protective against some cancers emerged in the 1990s [37]. Since the 1990s, a number of studies tried to understand what the role of plant foods was in the prevention of various types of cancer, including breast cancer, but despite this, evidence in support of this statement are inconclusive.

In 1992, Block *et al* reviewed 156 studies and concluded that ‘for most cancer sites, people with low fruit and vegetable intake experience approximately twice the risk of cancer compared to those with a high intake, even after checking for potentially confounding factors’. The risk decreased for cancers of the mouth and pharynx, oesophagus, stomach, colorectum, and lung. Breast cancer risk was not affected [38].

Other large prospective studies have investigated whether high intakes of fruit and vegetables might be associated with a reduced risk of breast cancer, but the results are close to null. In the women’s health initiative randomised trial, an increase in fruit and vegetable intake of 1.1 servings per day, did not cause a significant change in the incidence of breast cancer after 8 years [39].

Accordingly, in the 2007 WCRF/AICR report, it was stated that the evidence for an association between fruit and vegetable intake and breast cancer risk was too limited or inconsistent for a conclusion to be made. However, a more recent meta-analysis, which reviewed fifteen prospective studies, revealed a lower risk of breast cancer for the highest versus the lowest intake of fruit and vegetables combined, but when fruit and vegetable were separated, data were significant only for fruit consumption [40]. Interestingly, this study highlights that dietary changes during follow-up can obscure associations between dietary intake and disease risk if dietary intake is only assessed at baseline; in support of this statement, one of the studies analysed in this systematic review reported a RR of 0.59 (95% CI: 0.40–0.87) for high versus low intake of fruit, berries, and vegetables among women without a dietary change in the past, while there was no association among people who reported that they had changed their dietary intake, RR = 1.26 (95% CI: 0.63–2.55). This finding supports the hypothesis of epigenetic modulation through nutrition in early life and their role in the prevention of chronic diseases.

In addition, the results seem to be clearer when they are analysed in combination with the different type of tumours, in particular the expression of the ER. In this matter, the Pooling Project of Prospective Studies of Diet and Cancer analysed 20 studies and reported that only for oestrogen receptor-negative (ER−) breast cancer, there was a statistically significant inverse trend for total fruit and vegetable consumption; in this case, when intakes of fruit and vegetables were examined separately, statistically inverse associations were observed only for vegetable intake [41]. It should be stressed that one of the main difficulties in these kinds of studies is the assessment of food intake, highly variable because of the different methods and nutrient database utilised [36]. To improve knowledge in this issue, exposure assessment can be measured using biomarkers of fruit and vegetable intake, such as circulating carotenoids. A pooled analysis of eight cohort studies published in 2012 analysed the associations between circulating carotenoids and breast cancer risk. Results showed statistically significant inverse associations between circulating levels of total carotenoids and breast cancer risk. Associations were generally stronger among women of a healthy weight and for ER-negative tumours [42]. Several potential mechanisms may explain the inverse association between fruit and vegetables and breast cancer risk. These foods are important sources of fibre which may prevent breast cancer by binding oestrogens and reducing reabsorption of oestrogens in the colon. Moreover, fruit and vegetables are good sources of various phytochemicals, such as carotenoids, glucosinolates, indoles, and isothiocyanates, which may prevent breast cancer by inducing the activity of detoxifying enzymes, reducing oxidative stress, inflammation and changing the epigenome [43]. A high intake of fruit and vegetables may also reduce the risk of overweight/obesity, which is an important risk factor for postmenopausal breast cancer [10].

## Presumptions about soy products and the risk of breast cancer

Soybeans and its products have been a staple part of the Asian diet for centuries; they are the predominant source of isoflavones, which belong to the family of phytoestrogens. Genistein and daidzein are the most known and studied phytoestrogens. Laboratory data show that isoflavones have a wide range of biological actions, including the growth inhibition of breast cancer cell lines [44]. From this, much has been said about their role in carcinogenesis but what we know about the role of soy consumption in breast cancer risk in human is unclear.

Since 1991, after the publication on Lancet by Lee HP and colleagues [45], many observational studies reported that soy intake may lower breast cancer risk, but these findings are highly variable. On the contrary, due to the apparent oestrogenicity of genistein, women diagnosed with breast cancer have frequently been advised to avoid it. So, it would be useful to make sense of this issue.

In 2008, Wu *et al* conducted two separate meta-analyses of studies carried out in Asian and Western populations. A meta-analysis of eight studies showed that Asian women consuming the highest amount of dietary isoflavones had a reduction in breast cancer risk, as compared with those with low consumption of isoflavones. When stratified by the amount of soy consumed, a dose–response relationship was reported with a statistically significant trend of decreasing risk with increasing soy food intake, translating to a 16% risk reduction per 10 mg of daily isoflavone consumed. In contrast, a meta-analysis of 11 studies of women eating Western diets found no association between isoflavone intake and breast cancer risk [46]. A more recent meta-analysis tried to discriminate soy isoflavone intake and breast cancer risk for menopausal status with similar results [47].

Studies that investigate the relationship between soy food intake after the diagnosis of breast cancer and health status reported a slightly protective effect especially among the Asian population [48, 49] confirming the results collected so far.

There are different mechanisms that try to explain these various outcomes: first, the role of diet in the epigenetic modulation especially during early life; indeed, the protective role of soy is more evident in Asian countries, where soy products are the basis of the typical diet since childhood. In support of this statement, several case–control studies found that soy intake during childhood or adolescence affects breast cancer risk in adulthood [50]. Similar studies also show that lifetime soy consumption at a moderate level may prevent breast cancer recurrence through mechanisms that change the biology of tumours; in particular, women who consumed soy during childhood are more likely to develop breast cancers that express significantly reduced Human epidermal growth factor receptor-2 levels (hEGFR2). Another mechanism that may explain the differences between populations could be related to the intestinal microbial transformation of phytoestrogen, since the metabolism of isoflavone can vary greatly between individuals. Equol (EQ), a metabolite of daidzein produced in the intestine, is absorbed more efficiently and has higher oestrogenic activity then daidzein, but it was found that more than half of the adult population did not have a gut microbiota able to convert daidzein into EQ. This phenomenon has led to the classification of subjects into ‘EQ producers’ or ‘non-EQ producers’ [51]. The prevalence of EQ producers appears to be higher in Japanese than in Caucasian women, which might explain the additional benefits conferred on Japanese women in terms of reduced breast cancer risk [52]. It is possible that the consumption of soy since early life can promote a suitable gut microbiota; however, there is a lack of data to support this hypothesis. In summary, there is no evidence to advise against soy food consumption for the female population or women diagnosed with breast cancer. It is important to have a different approach with isoflavones supplements, as they provide a greater concentration of phytoestrogen, which indicates that interactions with biological mechanisms can be hazardous to health.

## Presumptions about milk, dairy products, and the risk of breast cancer

Milk and dairy consumption has, over time, been suspected of playing a role in the development of breast cancer. The hypotheses that have been put forth to suggest an increased cancer risk associated with milk consumption include overall high dietary fat content, contaminants in milk and hormones contained in milk such as oestrogens and IGF-1 [53].

IGFs are also self-produced by the human body and have both immediate and long-term effects on various cellular activities, for example impact on cell proliferation, differentiation, migration, and survival. The mitogenic and survival function of IGFs is not only observed in normal mammary cells, but also in breast cancer cells. Additionally, in some cases the IGF-1 receptor is overexpressed and highly activated in breast cancer tissue compared with normal or benign tissue. What is important to emphasise is that the actions of IGFs can be modulated by interaction with a family of six insulin-like growth factor-binding proteins (IGFBPs) and their functions at the cellular level are not fully understood [54].

In a consistent systematic literature review that summarised and quantified the current findings on milk and dairy products consumption and its effect on the serum concentration of IGF-1, 10 cross-sectional studies showed statistically positive correlation between milk consumption and the circulating IGF-1 level and randomised controlled trials indicated that the circulating IGF-1 level was significantly higher in the milk intervention group. However, only three out of 12 studies reported a statistically positive correlation between dairy products consumption and IGF-1 serum concentration [55].

Many epidemiologic studies that examined the association between diary product consumption and the risk of breast cancer have produced conflicting results, with inverse, positive or null associations reported [56].

A former meta-analysis published in the British Journal of Cancer concerning dietary fat and breast cancer risk, reported a positive association between milk and cheese consumption and the incidence of breast cancer [57].

However, a more recent similar meta-analysis showed a significant dose–response inverse association between all dairy food, but not milk, consumption, and breast cancer risk [58].

A single study was published recently with regard to the association between milk and dairy consumption and recurrence and mortality after breast cancer diagnosis.

In this cohort, 1,893 women diagnosed with early-stage invasive breast cancer were included, and dietary habits were assessed with a Food Frequency Questionnaire. Consistent with previous literature, the results showed no overall association between dairy intake and recurrence or breast cancer–specific survival. However, high-fat dairy intake was related to poorer overall survival in long-term breast cancer survivors [59].

Evidence of the relationship between milk and dairy products and the risk of cancer points in different directions, thus making it difficult to draw a conclusion; the best advice towards milk, dairy products, and breast cancer prevention is to consume these foods in line with the recommendations for a healthy diet, which favours low-fat dairy products in order to restrict the intake of saturated fats.

## Discussion

One of the main limitations in the field of nutritional science is that food and nutrients are not consumed in isolation and, from an epidemiological point of view, form a complex network of correlated influences. Therefore, it is difficult to study dietary patterns, which simultaneously reflect these exposures [60]. Moreover, prospective studies are required to improve dietary assessment instruments and develop new biomarkers, particularly recovery biomarkers of dietary intake.

Similarly, the stratification of breast cancer by specific characteristics should be further considered, particularly the individual metabolism, genetics, receptor status (oestrogen receptor, progesterone receptor, human epidermal growth factor receptor-2), substantial decline of ovarian hormones after menopause and other molecular classifications.

It is possible that the beneficial effects of dietary exposures are restricted to subgroups of women defined by specific genetic characteristics; therefore, future studies of gene-diet interactions will have to take into account genetic polymorphism when associations between dietary exposures and breast cancer risk are analysed.

In addition, most of the evidence in the literature has been obtained from studies that have evaluated diet during midlife and later, whereas the food exposures during menarche and first pregnancy may be more important in the development of breast cancer [61]. This is an example of the importance of the critical time period of exposures and of the long-term diet effects of diet and other lifestyles on the epigenetic mechanisms.

There are few strong reproducible and convincing associations between dietary patterns and breast cancer incidence and/or mortality. This fact is reflected in the conclusion of the last Continuous Update Project (CUP) on breast cancer by WCRF, which attributes only to alcoholic drinks and body fatness a convincing role as dietary factors that increase breast cancer risk [31].

The lack of clear evidence for an effect of other nutritional parameters, such as the use of fruit and vegetable, fibre-rich diet and reduced red-meat consumption, is reflected in the absence of agreed procedures for health professionals; as a result, despite the ‘healthy diet and lifestyle’ suggested by WCRF/AICR and other cancer agencies, healthcare providers do not have a landmark for breast cancer prevention [7].

According to Teegarden and colleagues, the best study that could provide direct evidence of the causal relationship between diet and cancer and to assess whether the adherence to the WCRF/AICR recommendations may modify cancer risk in a population, would be a randomised controlled trial. However, the methodological difficulties in applying, maintaining and verifying the dietary intervention for a sufficient period of time, make this kind of study infeasible.

However, indirect but quite robust evidence can also be obtained from observational prospective studies, such as the European Prospective Investigation into Nutrition and Cancer (EPIC). EPIC is a multicentre prospective study aimed at investigating the complex relationships between nutrition and various lifestyle factors and the aetiology of cancer and other chronic diseases. The study was initiated in 1993 in nine European countries and the field work was completed in 1998 with the inclusion of 484,042 subjects. All had provided questionnaire data [62]. Romaguera *et al* in 2012 constructed a score based on WCRF/AICR recommendations using EPIC questionnaires data from 386.355 participants. After a median follow-up time of 11 years, the results suggest that adherence to the WCRF/AICR recommendations for cancer prevention on diet, nutrition, physical activity and weight management, is associated with a lower risk of developing most types of cancer, including breast cancer [63]. This confirms that ‘healthy diet and lifestyle’ are important tools for the prevention of cancer.

Moreover, after a diagnosis of cancer, individuals are often motivated to change their diet, exercise habits, and other lifestyles. Many are also interested in learning more about dietary supplements and nutritional complementary therapies to manage persistent symptoms of disease or treatment [64].

For these reasons, it should be a primary goal of the International Institutions to design a ‘Breast Cancer Survivorship Care Plan’ considering the necessity of nutritional support for secondary prevention [65].

This plan can be targeted to meet individual needs of each patient, help improve survival and the quality of life [66].

## Conclusion

As breast cancer remains a significant scientific, clinical and social challenge, establishing a multidisciplinary research consortium is essential to demonstrate the real power of lifestyle interventions in order to reduce the risk of developing breast tumours in women. More efforts should be made to promote models of innovative approaches for preventing breast cancer, reducing the side effects of treatment and increasing survivorship.
